# Epidemiology of ocular trauma in limited-resource settings: a narrative review

**DOI:** 10.3389/fmed.2025.1585527

**Published:** 2025-08-25

**Authors:** Jessica Pelletier, Kakande Reagan, Sarah McLeod, Noah Kronk, Kamoga Dickson, Kyle Ohman, Matthew Santos

**Affiliations:** ^1^Department of Emergency Medicine, University of Missouri-Columbia, Columbia, MO, United States; ^2^Department of Medicine, Mbarara University of Science and Technology, Mbarara, Uganda; ^3^Department of Emergency Medicine, Makerere University School of Medicine, Kampala, Uganda; ^4^Department of Ophthalmology and Visual Sciences, Scheie Eye Institute, University of Pennsylvania, Philadelphia, PA, United States

**Keywords:** ocular trauma, emergency medicine, limited resource, low resource settings, low-income and middle-income countries, epidemiology

## Abstract

Ocular trauma disproportionately impacts low- and middle-income countries (LMICs) and contributes significantly to blindness and disability in these settings. While numerous publications address the epidemiology of ocular trauma in limited-resource settings, there are no systematic reviews, meta-analyses, or large-scale review articles investigating this topic further. In this article, the authors summarize, compare, and contrast the extant literature on ocular trauma in LMICs. With this synthesis of the available data, the article aims to identify commonalities and potential targets for systemic change in preventing ocular injury and its associated morbidity. The authors seek to highlight modifiable risk factors which may be addressed by providers, health care systems, government agencies, and employers alike.

## Introduction

Ocular trauma is a significant global public health concern, contributing to a substantial burden of visual impairment and blindness, particularly in low-resource settings. According to the World Health Organization (WHO), approximately 43 million people worldwide are blind, with an additional 295 million experiencing moderate-to-severe visual impairment (1). The disabling nature of blindness extends beyond personal hardship, leading to lost wages, diminished productivity, and an increased economic burden on families and communities. Ocular trauma is a leading cause of unilateral vision loss, with over 55 million eye injuries reported globally each year. Among these cases, approximately 19 million result in at least partial permanent vision loss in one eye, while 1.6 million people suffer complete blindness due to their injuries (2). Additionally, ocular trauma is a frequent reason for emergency department (ED) and outpatient visits worldwide with hospitalization rates ranging from 4.9 to 89 per 100,000 cases (3, 4).

The impact of ocular trauma is particularly severe among vulnerable populations, such as children. In low-income countries (LICs), 60%–80% of blind children die within 1–2 years of becoming blind (5), reflecting the devastating consequences of visual impairment in settings with limited access to healthcare and social support. Contributing factors may include malnutrition, decreased mobility, limited access to education, and an increased risk of accidents and neglect, all of which exacerbate the challenges faced by visually impaired children in resource-poor environments. Despite the critical need for intervention, there remains a lack of comprehensive epidemiological studies analyzing ocular trauma across multiple regions and healthcare systems. Identifying common causes of ocular injury in resource-limited settings is essential for developing standardized prevention measures and enhancing public health education.

This narrative review aims to synthesize epidemiological data on ocular trauma across diverse geographic regions, analyze demographic and trauma-related characteristics, and identify high-risk populations, including children, women, and the elderly. Additionally, we will explore complications associated with ocular trauma, prevention strategies, treatment approaches, and visual rehabilitation efforts. By consolidating existing knowledge, we hope to provide objective reference data for ophthalmologists and public health professionals to improve ocular trauma prevention, management, and long-term patient outcomes.

## Materials and methods

The authors searched PubMed, Google Scholar, and the University of Missouri-Columbia library database for articles using the search terms “ocular trauma,” “emergency medicine,” and “epidemiology” combined with each of the following: limited resource, low resource settings, LICs, middle-income countries. The search was conducted from database inception to January 2025. Hand-searching of personal files was conducted, and reference sections of the retrieved articles were also utilized to identify additional articles for review. Authors evaluated systematic reviews and meta-analyses, randomized controlled trials (RCTs), prospective and retrospective studies, survey studies, case reports and series, and other narrative reviews, with preference for systematic reviews and meta-analyses, when available. References cited by selected articles were also reviewed, and only English-language articles were considered for review. Articles excluded were those (1) discussing the epidemiology of ocular trauma in high-income countries (HICs) and (2) those for which full-text articles could not be located.

The World Bank classification system was utilized to categorize the economies of countries in order to exclude studies from HICs. The World Bank uses 2023 gross national income (GNI) per capita to classify countries as low-income (≤$1,145), lower middle-income ($1,146–$4,515), upper middle-income ($4,516–$14,005), or high-income (>$14,005) (6). GNI per capita refers to the overall net income of a country distributed by person (7). Categorization of countries into regions was also based on the World Bank classification system (6).

## Results

**FIGURE 1:**
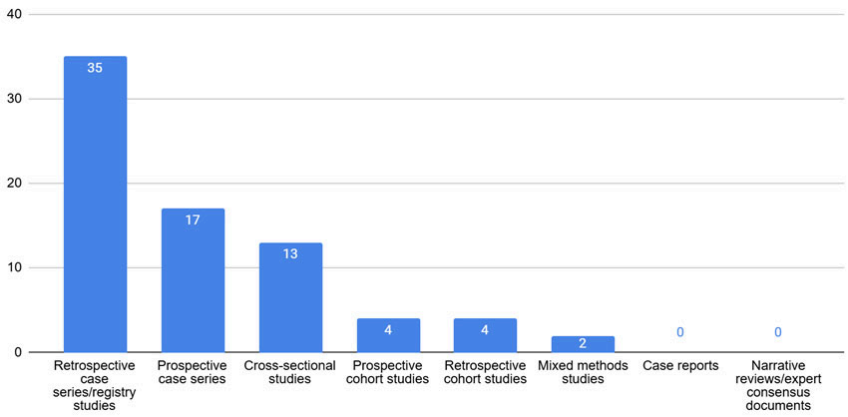
Graphic demonstrating the numbers and types of articles included in this narrative review.

75 articles were selected for inclusion in this narrative review. Among these, there were 0 systematic reviews and meta-analyses, 0 RCTs, 13 cross-sectional studies, 4 prospective cohort studies, 17 prospective case series, 4 retrospective cohort studies, 35 retrospective case series/registry studies, 2 mixed methods studies, 0 case reports, and 0 narrative reviews or expert consensus documents (Figure 1).

**FIGURE 2:**
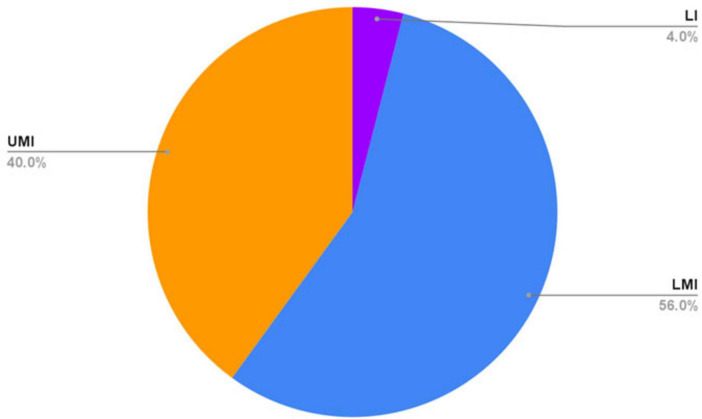
Economic classifications by the World Bank of countries in which included articles were published. LI, low-income; LMI, lower middle-income; UMI, upper middle-income.

**FIGURE 3:**
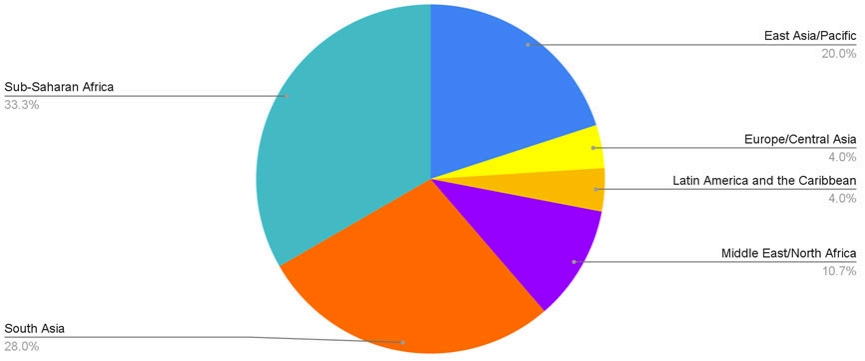
Article breakdown by region of publication.

3 articles were conducted in LICs, 42 were conducted in lower middle-income countries, and 30 were conducted in upper middle-income countries (Figure 2). 15 studies were conducted in East Asia and the Pacific; 3 studies in Europe and Central Asia; 3 studies in Latin America and the Caribbean; 8 studies in the Middle East and North Africa; 21 studies in South Asia; and 25 studies in sub-Saharan Africa (Figure 3). 21 articles studied only pediatric patients (8–28), and 7 articles only studied adult patients (29–35). Details regarding all articles reviewed can be found in Supplementary Table 1.

Males of all ages were noted to experience more ocular trauma as compared with females. By and large, young male adults of working age had the highest incidence of ocular injury across studies. There was some variation in the exact ages of those with the highest incidence of ocular trauma. In most pediatric studies, the 6–10 years age group tended to experience the highest incidence of ocular trauma (9, 17, 18, 25, 28).

Articles evaluating ocular trauma patients in the outpatient setting or the emergency department (ED) reported a majority of injuries to be closed-globe in nature (14, 22, 23, 27, 36–40). 16 articles (3 prospective and 13 retrospective) evaluated patients admitted for ocular trauma. Open globe injury (OGI) was noted in the majority of patients admitted for ocular trauma across studies (9, 15, 20, 21, 34, 41–45). Presenting visual acuity (VA) was noted to predict final VA across multiple studies (26, 27, 46–49). Patients with OGI were consistently noted to experience worse VA outcomes as compared with closed-globe injury (CGI) (23, 27–29, 44, 50–53). Those with delays in seeking ophthalmologic specialty care were also noted to have worse VA outcomes (54), as were those with delays in surgical management for OGI (55).

Home was the most common location of occurrence of ocular trauma in 15 studies (9, 10, 19, 21–23, 27, 28, 32, 33, 37, 42, 46, 47, 56, 57) compared to the workplace in 8 studies (41, 48, 53, 58–61, 62). Road traffic accidents (RTA) were the leading cause of injury in 4 studies (32, 38, 63, 64). Assault was the most common mechanism of injury across studies conducted in Nigeria and South Africa (30, 31, 33, 39, 57, 65), and domestic violence against women as a common cause of ocular trauma was noted in 3 studies (32, 40, 66). Children were commonly injured during play or sports (9, 16, 17, 27, 67, 68, 69). Implements causing injury varied widely across studies, but sharp objects were noted as the implement of injury in numerous pediatric studies (8, 9, 15, 24, 25, 58). Fireworks were noted to be a significant cause of injury in 4 studies, 1 conducted in Iran (8), 1 conducted in India (19), and 2 conducted in China (60).

Multiple studies noted that patients suffering from ocular trauma were not wearing eye protection at the time of injury, even in situations where eye protection would be considered essential (19, 38, 61, 70, 71). One study noted that individuals with higher levels of education were more likely to use eye protection (72). Pediatric patients suffering from ocular trauma were often noted to sustain their injuries in situations where parental supervision was poor (8, 10, 14). One study noted a lack of seatbelt use in the majority of cases of ocular trauma sustained during RTA (52). Two studies noted an association between ocular trauma and alcohol use (33, 34).

## Recurring prevention recommendations

Throughout the studies reviewed, there were several recurring prevention recommendations identified: emphasizing the importance of community education, workplace safety, healthcare improvements, legislative actions, and societal changes. Public education efforts should focus on raising awareness about ocular trauma prevention, encouraging early presentation for injuries, and utilizing media and school-based campaigns (9, 19, 57, 58). Increased parental supervision, particularly during sports and at home, along with proper storage of sharp objects and toxic chemicals, is essential to reduce childhood injuries (8, 9, 14, 16–19, 22–24, 26, 28, 42, 58, 59, 75), as is restricting children’s access to fireworks (8, 23, 43, 60). Workplace safety measures should include mandatory protective eyewear, comprehensive employee training–even for temporary workers–and stricter enforcement of federal safety regulations (22, 29, 39, 44, 45, 48, 49, 51, 56, 58–63, 74, 76–78). Recreational safety should be enhanced through the use of protective eye gear in high-risk sports (9, 49, 78, 79), financial support for low-income athletes (to finance the purchase of such eye gear) (78), improved playground protections (56), and stricter regulations on seatbelt (9, 23, 43, 52, 61), car seat (9), helmet use (38, 51, 61), and cell phone restrictions while driving (64). Strengthening healthcare systems through better training for healthcare workers (51, 54, 73), outreach to rural communities (26, 35, 60), and improved access to emergency ophthalmologic care (18, 20, 27, 33) is also critical. Legislative actions should focus on enforcing child labor laws (8), strengthening regulations for protective eyewear in workplaces (59), enforcement of traffic laws (38, 63), restricting access to fireworks and firearms (8, 19, 23, 43, 44, 60), and regulating the sale of prescription eye drops (73). Public works should aim to improve road markings and maintenance to ensure traffic safety and reduce the frequency of RTAs (52, 64). Additionally, societal efforts to curb alcohol abuse (30, 31), prevent interpersonal violence (30, 31, 33, 80), and promote gender equality (31) could play a crucial role in reducing ocular trauma incidents.

## Discussion

This narrative review provides an overview of published literature on ocular trauma epidemiology in limited-resource settings. The incidence of ocular trauma is increasing over time and is associated with significant morbidity, imposing a serious public health problem (81). Although the risk of ocular trauma varies significantly across different age groups in LMICs, a consistent finding was that men ages 20–40 faced the highest risk of ocular injuries due to occupational hazards, engagement in high-risk activities, and limited access to protective measures (50). Men working in manual labor sectors like construction and welding, are commonly exposed to flying debris, intense light, and chemicals; yet, protective eyewear use remains low (53.71%) due to inadequate training and enforcement (82). Limited access to personal protective equipment (PPE) and poor adherence to its use in many work settings in LMICs further exacerbates the burden of ocular trauma. The World Health Organization’s World Report on Vision emphasizes that inadequate access to eye care services and protective measures disproportionately affects individuals in LMICs (83) (particularly those who are financially disenfranchised) (84), contributing to a higher incidence of preventable vision impairment and blindness (83).

Both HICs and LMICs report a higher incidence of ocular trauma among men compared to women, a disparity largely attributed to the nature of male-dominated activities and occupational exposure (81). Men are more likely to engage in high-risk professions such as construction, manufacturing, mining, and agriculture, where the likelihood of eye injuries from flying debris, sharp objects, and hazardous chemicals is significantly increased. Additionally, men frequently participate in contact sports and recreational activities with a greater potential for facial and ocular trauma, including football, boxing, and martial arts. Furthermore, studies indicate that men are more frequently involved in physical altercations, interpersonal violence, and high-risk behaviors such as unsafe driving and hazardous work practices, all of which contribute to an elevated risk of eye injuries. The combination of occupational exposure, recreational risks, and behavioral tendencies underscores why men are disproportionately affected by ocular trauma across diverse socioeconomic settings.

In rapidly industrializing countries, the increase in manufacturing and construction activities has led to a notable increase in work-related ocular injuries. This trend is largely due to the expansion of construction and manufacturing industries, where workers are frequently exposed to eye hazards such as flying debris, chemical splashes, intense light flashes, and radiation. This narrative review highlights an increased burden of ocular trauma in the rapidly industrializing world as demonstrated by several studies, including a recent study by (85) Rapid industrialization often outpaces the establishment and enforcement of occupational safety regulations, leading to inadequate use of PPE. Several studies have shown inconsistencies in the use of PPE among factory and farm workers in LMICs, which increases their risk of ocular injuries (86, 87). This lack of adherence to PPE usage, coupled with insufficient safety training, contributes significantly to the higher incidence of ocular trauma in these settings.

Work-related ocular trauma is more prevalent in developing countries compared to developed nations, with young males being the most heavily affected age group in both settings (81). In many low-resource settings, the use of personal protective equipment (PPE) among manual laborers and factory workers remains limited. Many casual laborers continue to work without essential protective gear, such as goggles and eye shields, which significantly increases their risk of ocular injuries. This lack of PPE use is largely due to its inadequate availability, limited awareness of its importance, and insufficient training on proper usage (74).

In contrast, developed countries enforce strict PPE regulations, making it mandatory for all factory workers to wear protective eyewear. This is further reinforced by greater awareness and comprehensive training on workplace safety, significantly reducing the incidence of occupational-related ocular injuries. Additionally, the use of automated engineering systems and controlled work environment limits human exposure to hazardous materials, lowering the risk of ocular injuries in HICs. Well-developed occupational health programs and strong employee legal frameworks holds employers accountable, ensuring that they provide PPEs for workers efficiently to minimize their exposure to hazardous materials (88).

Open globe injuries were notably prevalent among hospitalized ocular trauma patients in the included studies. The complexity and severity of these injuries necessitate immediate and specialized medical intervention, often requiring hospitalization for surgical repair and extensive postoperative care. A population-based survey analyzing work-related ocular injuries found that 46% of hospitalized cases were due to OGIs, underscoring their severity and the need for inpatient management (89). The delay in seeking care, as noted in multiple studies, may contribute to the progression of injury, necessitating longer durations of hospitalization and higher resource utilization.

This review highlights variations in injury patterns between rural and urban settings, reflecting a difference in the occupational and environmental risks leading to ocular trauma. In rural areas, agricultural activities are the primary contributors to ocular trauma, with injuries resulting from plant matter, farming tools, and animal-related accidents. Studies have found that agricultural activities account for the majority of ocular injuries in rural settings, with corneal abrasions and foreign bodies from plant debris being the most common causes (29). These injuries often occur due to a lack of protective eyewear and manual farming practices that increase exposure to environmental hazards. In contrast, studies from highly urbanized areas reported a higher prevalence of ocular injuries caused by sharp, man-made objects, such as metal fragments, glass material, and machinery-related accidents. The greater industrialization of urban workplaces explains this difference in injury mechanisms. The risk of ocular trauma in industrialized settings is further heightened by inadequate enforcement of workplace safety regulations and the inconsistent use of protective eyewear (90). These differences in trauma mechanisms highlight the need for tailored preventive strategies. In rural settings, education on eye protection during farming activities and the provision of safety gear such as goggles could help reduce injuries from plant debris and tools. In industrial areas, stricter enforcement of safety regulations, the mandatory use of protective eyewear, and regular safety training could significantly lower the incidence of injuries caused by sharp objects. Addressing these setting-specific risks is crucial in minimizing the burden of ocular trauma across different environments.

Access to ophthalmologic services in LMICs is hindered by geographic, economic, and cultural barriers. Many rural residents live far from healthcare facilities and cannot afford to travel for ophthalmologic specialty evaluation. A critical shortage of ophthalmologists leads to overburdened facilities and long wait times, discouraging care-seeking. Additionally, limited awareness of eye health results in delayed treatment, and traditional beliefs often lead individuals to seek alternative remedies instead of professional care. Collectively, these factors lead to significant delays in accessing care among patients with ocular injuries, resulting in poor visual acuity outcomes.

A global standard for eye safety in high-risk occupations should be developed and enforced, regardless of the country’s income level. Protective eyewear should be mandatory for industries such as construction, agriculture, and manufacturing. Studies from China and India have found that workplace injuries are a major cause of ocular trauma, with many patients failing to use protective eyewear (71, 74). However, implementing such standards poses challenges, particularly in resource-limited settings, where governments may find providing eye protection cost-prohibitive (71). Collaborative efforts between governments, international organizations, and employers could help subsidize protective equipment and encourage its regular use. Additionally, eye protection must be tailored to specific workplace hazards to be effective. For example, agricultural workers face a significantly higher risk of ocular trauma than manual laborers in Bosnia and Herzegovina (85). This study further emphasizes the need to identify the primary causes of ocular injuries across various work environments and develop specific prevention strategies.

Community-based educational campaigns are vital for reducing the incidence and severity of ocular trauma. These programs should prioritize parental supervision and keeping potentially dangerous objects out of reach of children, as most pediatric ocular trauma occurs at home or during unsupervised play (8, 10, 14). In other settings, childhood labor and exposure to dangerous objects such as sharp tools or fireworks, are significant risk factors (8, 17). In countries such as Nigeria and Bangladesh, the use of home remedies and traditional healing rather than seeking formal medical care increases the likelihood of experiencing irreversible poor visual outcomes (66, 70). Studies suggest that early presentation to eye care facilities significantly improves visual outcomes (36, 54). Thus, campaigns should be initiated to emphasize the importance of promptly seeking professional care in the event of ocular trauma.

Two studies conducted in Eritrea noted landmine explosions as a significant cause of blindness among children (12, 13). In LMICs experiencing war, blast injuries and assault account for the majority of the ocular injuries. Shrapnel, debris and radiation from the explosives cause direct injury to the globe thus affecting vision. Additionally, chemical agents from explosives can cause severe ocular surface damage, leading to long-term visual impairment. Gunshot wounds to the head frequently result in ocular injuries due to the proximity of the eyes to the trajectory of bullets. Research has shown that survivors of gunshot wounds to the head suffer long-term visual impairment due to direct ocular trauma (91). In an effort to reduce ocular trauma secondary to conflict and violence, countries currently at war or recovering from conflict should implement protective measures to prevent trauma from explosives. One method for reducing injury would be marking hazard zones for blast injuries to reduce pedestrian traffic and accidental injuries. In countries with significant systemic violence issues such as Nigeria and South Africa, assault and chemical attacks are leading causes of bilateral ocular trauma (30, 31, 33, 39, 57, 65). Implementing stricter gun control and reducing the availability of harmful chemicals, such as car battery acid, could help prevent such injuries (65).

A number of studies have noted that domestic violence was a significant cause of ocular injury among women (32, 40, 66), and these authors emphasize the benefits of supporting domestic violence victims. LMICs experience a discrepantly high burden of domestic violence compared to high-income countries (92). Providing shelters, alternative housing solutions, and improving access to support services for women could help reduce these preventable injuries (93).

To complement prevention strategies, healthcare systems must be equipped to manage ocular trauma effectively. Various measures can be implemented, such as training of healthcare workers on how to manage ocular injuries and providing urgent referrals for specialized care. In some studies conducted in Tanzania and Nigeria, mismanagement at lower-level facilities and delayed referrals were noted, emphasizing the need for regular training of healthcare workers without specialty training in ophthalmology (51, 73). Cost and transportation barriers, combined with the limited availability of ophthalmologic services outside of large academic medical centers all contribute to the delayed presentation of ocular trauma (54, 75). Both government and private sectors must work to provide affordable and accessible eye care services in order to reduce the societal burden of vision impairment secondary to ocular trauma.

As suggested by authors based in China and India, the introduction of regional or national ocular trauma registries could provide valuable data to guide public health interventions and resource allocation (41, 71). The International Globe and Adnexal Trauma Epidemiology Study (IGATES) ophthalmic trauma registry currently exists, but engagement across countries is not universal (94). Preventing ocular trauma requires collective action from governments, healthcare systems, communities, and international organizations. By implementing safety standards, educating the public, reducing conflict-related injuries, and strengthening healthcare systems, the burden of ocular trauma in low-resource settings can be significantly reduced. A summary of ocular trauma prevention recommendations is included in Table 2.

**TABLE 1:** Key differences in ocular trauma between adult and pediatric populations based on the articles reviewed.

Key difference category	Children	Adults
Delay in presentation	Due to distance to care, lack of parental awareness, or use of traditional remedies (18, 28)	Due to attempting self-treatment first, injuries occurring on the weekend, or seeking non-specialist care first (70, 73)
Location and timing of injury	Injuries usually occurred in the home and during unsupervised play (8–10, 19) Injuries more commonly occurred during vacations and celebrations* (17, 26)	Injuries usually occurred in the workplace, particularly in agricultural, construction, and manufacturing settings (29, 74)
Mechanism of injury	Usually sharp objects, sporting equipment, or fireworks (8, 18, 25) Blast injuries were a notable cause in areas impacted by war (12, 13)	Usually occupational (71, 74) Assault was a notable cause, particularly in association with alcohol misuse (30, 33)

*Often due to exposure to fireworks.

**TABLE 2:** Ocular trauma prevention recommendations.

Category	Recommendations
Community education	• Encourage early presentation for injuries (19, 57, 58)
	• Increase parental supervision
	• Properly store sharp objects and chemicals (8, 9, 14, 16–19, 22–24, 26, 28, 42, 58, 59, 75)
	• Restrict access to fireworks (8, 23, 43, 60)
	• Use protective eyewear for sports (9, 49, 78, 79)
Healthcare improvements	• Expand emergency ophthalmologic care (18, 20, 27, 33)
	• Train healthcare workers in ocular injury management (51, 54, 73)
Legislative actions	• Enforce traffic laws (38, 63); mandate seatbelt, car seat (9, 23, 43, 52, 61), and helmet use (38, 51, 61)
	• Improve playground (56) and road safety (52, 64)
	• Outlaw child labor and enforce child labor laws (8)
	• Regulate prescription eye drop sales (73)
	• Restrict cell phone use while driving (64)
	• Restrict firework and firearm access (8, 19, 23, 43, 44, 60)
Societal changes	• Promote gender equality (31)
	• Reduce alcohol misuse (30, 31) and interpersonal violence (30, 31, 33, 80)
Workplace safety	• Educate employees on ocular injury prevention
	• Mandate protective eyewear
	• Strict enforcement of federal safety regulations (22, 29, 39, 44, 45, 48, 49, 51, 56, 58–63, 74, 76–78)

### Strengths and limitations

This review includes studies from a wide range of LMICs, providing a comprehensive understanding of ocular trauma across various socioeconomic and healthcare settings, highlighting common patterns, context-specific challenges, and offering a broad scope of analysis. The review also draws attention to low-resource settings, where the burden of ocular trauma is often underreported and under-researched.

Unlike a systematic review, the narrative review approach lacks a structured methodology for study selection, which increases the risk of omitting relevant literature and introducing selection bias. Studies are included from a wide range of socioeconomic contexts and levels of industrialization, making direct comparisons challenging. The absence of data from North America limits the global applicability of the findings and may overlook regional variations in ocular trauma epidemiology. Furthermore, the differences in study designs ranging from community surveys to hospital-based case series affect the consistency and comparability of results. The review also involved retrospective studies, which rely on patient interviews, making them prone to recall bias.

Authors of the included studies used varying definitions of visual impairment and blindness across studies hindering direct comparisons and analytical insights. Many low-resource settings rely on paper-based records, leading to incomplete datasets that may underestimate the true burden of ocular trauma. Finally, several studies failed to include injury prevention recommendations, reducing the ability to identify context-specific solutions.

### Implications and future directions

This review can inform governments and employers to collaborate and enforce workplace safety regulations, ensuring that protective eyewear is both available and used, especially in high-risk occupations. It can also inform community outreach programs to focus on activities like injury prevention, emphasizing the importance of parental supervision, prompt medical attention, and avoiding harmful practices like home remedies which can reduce the injury related ocular trauma. Training programs for healthcare workers and the development of affordable, accessible eye care services are essential for improving trauma outcomes. Establishing uniform definitions for visual impairment and consistent data recording practices across studies will enhance comparability and the accuracy of future reviews. Future research should assess the effectiveness of educational campaigns, safety regulations, and healthcare system improvements in reducing ocular trauma incidence and improving patient outcomes.
