# Supplementary material for: Epidemiology of ocular trauma in limited-resource settings: a narrative review
Source: Front Med (Lausanne). 2025 Aug 25;12:1585527. doi: 10.3389/fmed.2025.1585527 (PMC12415046; doi:10.3389/fmed.2025.1585527)
Supplement: Supplementary file 1 [file Table_1.docx]

**Supplementary Table 1.** Studies included in this narrative review, stratified by location, study type, patient population, number of patients included, key findings, and recommendations from the authors for prevention of ocular trauma in their respective settings.

| **Study Author and Year** | **Study Location** | **World Bank Country Classification** | **Study Type** | **Patient Population** | **Number of Patients Included** | **Key Findings** | **Prevention Recommendations** |
| --- | --- | --- | --- | --- | --- | --- | --- |
| Addisu, 2011 [(71)](https://www.zotero.org/google-docs/?VYDc1J) | Butajira, Ethiopia | Low-income | Prospective case series | All consecutive patients with ocular trauma seen at an eye hospital over a 13-month period | 753 | Male predominance (75%)  Average age of patients was 27  Most common cause of injury = blunt objects  Penetrating trauma was responsible for majority of cases with VA < 6/60  More than half of patients had to travel a great distance (21 to > 100 km) for eye care, leading to delayed presentations | Community education to aid in ocular injury prevention and early presentation  Greater parental supervision |
| Ahmadabadi et al., 2010 [(10)](https://www.zotero.org/google-docs/?Sn41P3) | Tehran, Iran | Upper middle-income | Prospective case series | Pediatric patients (≤ 16 years) with OGI due to sharp injury mechanism presenting to the hospital over a 6-month period | 125 | Male predominance  Mean age was 8.5 years  Most common location where injury occurred = home  Most common causes of injury = knives and fireworks  Glasses contributed to injury in 2/3rds of patients who wore them | Educate parents about how to avoid eye trauma. The fact that these injuries didn’t happen at school suggests teachers and staff were more aware of these risks. Almost half occurred when kids were alone, suggesting the need for closer parental supervision  Childhood labor should be banned - work-related injury was a large cause of ocular trauma in older kids  Use glasses that are less likely to cause injury (i.e. polycarbonate, or soft contacts)  Avoid fireworks  Recommend use of plastic cutlery for children |
| Ajayi et al., 2014 [(68)](https://www.zotero.org/google-docs/?9hXwxM) | Ado-Ekiti, Nigeria | Lower middle-income | Prospective case series | All patients presenting to the hospital with ocular trauma over a 15-month period | 85 | Male predominance  Mean age was 32 years  Most common MOI = Occupational injury  Majority of patients were not wearing eye protection  The majority presented > 24 hours after injury  Most patients tried medications at home before arrival (the majority of these medications were antibiotics)  Majority of injuries were closed globe (87%) | None provided |
| Akudinobi and Nwosu, 2022 [(72)](https://www.zotero.org/google-docs/?eSsWI2) | Anambra, Nigeria | Lower middle-income | Cross-sectional study | Adult and pediatric destitute patients with visual impairment | 168 | Most common cause of blindness was glaucoma, followed by cataract, corneal disease, and trauma (9.5% of cases of blindness)  Prevalence of blindness 3x higher among the destitute than the general population | None provided |
| Al-Attas et al., 2010 [(73)](https://www.zotero.org/google-docs/?sTpJtX) | Moshi, Tanzania | Lower middle-income | Prospective mixed-methods study | All patients admitted with ocular injury over a 2-year period | 93 | Mean delay in treatment was 6.8 days  The majority of patients had visited a health facility within the first 48 hours of injury  Females experienced worse delays  Predictors of delay included weekend injuries, using topical eye medications, and visiting other health facilities prior to the ophthalmologic specialty center | Education of healthcare workers about appropriate management and referral for ocular trauma; regularly scheduled and recurring training to enhance ocular management skills  Better explanations from healthcare workers to patients about the importance of seeking follow-up ophthalmologic care in the setting of trauma  Community education regarding the serious nature of eye trauma  Better regulation of drugstores prescribing eye drops without appropriate training |
| Al-Bdour and Azab, 1998 [(11)](https://www.zotero.org/google-docs/?hyvsss) | Irbid, Jordan | Lower middle-income | Retrospective case series | Pediatric patients (≤ 16 years) with ocular trauma admitted to a teaching hospital over a 3-year period | 116 | Male predominance  6-10 year age group was most commonly affected  Most common injury location = home  Most common cause of injury = sporting/play  Most common MOI = Stones and sharp objects  Majority of cases were OGI  Complete loss of vision occurred in 13% of cases | Improved parental supervision at home  Storing sharp objects away from children  Community education regarding the importance of seatbelts and car seats while driving (though RTA was a very uncommon MOI in this study), as well as eye protection during sports  Using public media and informational flyers in schools to educate the public about the risk of blindness from ocular trauma |
| Bonsaana et al., 2015 [(37)](https://www.zotero.org/google-docs/?fnUqVb) | Tamale, Ghana | Lower middle-income | Retrospective case series | All patients presenting to the eye clinic of a teaching hospital with ocular trauma over a 1 year period | 361 | Male predominance  20–29-year age group was most commonly affected  Majority of injuries were CGI  Visual impairment occurred in a significant number of cases (32.6%) | Community education to aid in ocular injury prevention |
| Cai and Zhang, 2015 [(74)](https://www.zotero.org/google-docs/?IBxl5H) | Chongqing, China | Upper middle-income | Retrospective cohort study | All patients presenting to the hospital with ocular trauma over a 1-year period | 1055 | Male predominance  Average age of 37.5 years  Nearly half were work-related injuries  Mean age of those with work-related trauma was higher than for those with non-work-related trauma  Metal = most common cause of injury  Those working in manufacturing without protective glasses or safety training were more likely to suffer injury  Temporary workers are more likely to be injured  Peak incidence of injury during the summer and right before the end of the workday | Better training and protective measures for those in manufacturing to prevent injury  Even temporary workers need training to prevent injury  Make workers aware that injury risk is higher when fatigued (i.e. working too many hours or feeling hot) - take more breaks  Developing countries should have mandated reporting for preventable injuries |
| Cao et al., 2012 [(42)](https://www.zotero.org/google-docs/?hBlWCy) | Chaoshan Region, China | Upper middle-income | Retrospective cohort study | All patients admitted for ocular trauma at 3 hospitals over a 10-year period | 3644 | Male predominance  Children and young adults were the predominant patient population.  OGI = majority of cases, followed by CGI, chemical injuries, thermal injuries, and adnexal injuries  Workplace-related injuries more common than those occurring at home  9.5% presented after 24 hours | Public education regarding the importance of early presentation for eye injury  Establishment of a national collaborative registry to document all eye injuries. |
| Chaikitmongkol et al., 2015 [(31)](https://www.zotero.org/google-docs/?yoEjH9) | Chiang Mai, Thailand | Upper middle-income | Prospective case series | Adult patients (≥ 19 years) presenting to a university-based referral eye center with ocular trauma over a 15-month period | 97 | Male predominance  Majority did not use protective eyewear  59% lacked knowledge about it, 22% lacked equipment, 18% had both but didn't use it anyway  Agriculture was the most common profession among those injured  OGI = majority of cases  Nailing and motorized grass trimming were significantly associated with OGI | Use of protective Eyewear among workers, especially those conducting nailing or motorized grass trimming  Education regarding the risks of ocular injury, especially for agricultural and construction workers |
| Choovuthayakorn et al., 2020 [(59)](https://www.zotero.org/google-docs/?w79pQy) | Chiang Mai, Thailand | Upper middle-income | Retrospective case series | All patients admitted to the hospital with ocular trauma over a 1-year period | 249 | Male predominance  Age 40-60 had the highest incidence of ocular trauma  Most injuries were workplace-related  Similar percentages of OGI vs. CGI  Patients with CGI tended to present later  RTA was a major MOI for young people 15-40 years  For children, most common MOI = unintentional striking with a sharp wooden object | Greater safety education for parents, more supervision for children  Better driving education  Social media programs to raise awareness about the need for protective eyewear in the workplace |
| Dandona et al., 2000 [(75)](https://www.zotero.org/google-docs/?0YNkVv) | Hyderabad, India | Lower middle-income | Cross-sectional population-based study | 24 clusters of patients representative of the local population who presented for an interview and detailed ocular exam | 2522 (113 with ocular trauma) | 1/25 people in the general population were affected by ocular trauma, and 1/167 were blinded by trauma  OR for ocular trauma was higher for males (2.5)  OR for ocular trauma was higher (2.5) for laborers (compared with other types of workers)  Most common MOI was during play | Mothers and children of low SES should be targeted for injury prevention education |
| Dhasmana and Bahadur, 2012 [(63)](https://www.zotero.org/google-docs/?xe2BIV) | Dehradun, India | Lower middle-income | Prospective case series | All patients presenting to the hospital with ocular trauma over a 1-year period | 88 | Men were 2-fold more likely to suffer ocular trauma  Mean age 31.2  Most common cause of injury = RTA, followed by occupational injury  Of note - higher rate of RTA-induced ocular injury in their study than those performed in developed countries  Among those injured at work, those working in industrial occupations = highest risk  Women more commonly injured at home  17% presented > 48 hours after injury | Better education and more use of protective equipment to protect from injury at work  More strict reinforcement of traffic laws to prevent RTAs |
| Dulal et al., 2012 [(12)](https://www.zotero.org/google-docs/?3wZ2iW) | Pokhara, Nepal | Lower middle-income | Retrospective case series | Pediatric patients (< 16 years) presenting to the hospital with ocular trauma over a 1-year period | 554 | Male predominance  Ages 5-10 had the highest incidence of ocular trauma  Only 16% visited the hospital within 24 hours of injury  Most common location of injury = home | Improving literacy and health awareness |
| du Toit et al., 2013 [(32)](https://www.zotero.org/google-docs/?j0EYf1) | Cape Town, South Africa | Upper middle-income | Prospective case series | Adult patients (≥ 13 years) presenting with OGI over a 2-year period | 249 | Male predominance  Most common MOI = assault  The majority of cases were associated with ethanol use  Most victims knew their assailants as acquaintances or friends | Societal efforts to curb violence and alcohol abuse |
| Eballe et al., 2009 [(13)](https://www.zotero.org/google-docs/?QZeiRy) | Yaoundé, Cameroon | Lower middle-income | Prospective case series | Pediatric patients (6-15 years) presenting to a pediatric hospital with unilateral blindness | 1266 (60 with unilateral blindness) | The majority of cases of unilateral blindness (65%) were caused by ocular trauma | Public education on avoidance of ocular trauma |
| Gorleku et al., 2020 [(64)](https://www.zotero.org/google-docs/?phGeJI) | Cape Coast, Ghana | Lower middle-income | Retrospective case series | All patients receiving a head CT scan for an indication of trauma over a 2-year period | 1043 | Male predominance  14 types of NOI identified; IOFB most common  Most common MOI = RTA | Work to decrease the number of RTAs by improving education for drivers, removing vehicles that have broken down from the side of the road, better maintenance of roads, restricting cell phone use while driving |
| Grace et al., 2014 [(65)](https://www.zotero.org/google-docs/?3bYD9u) | Kwara State, Nigeria | Lower middle-income | Retrospective case series | Patients with bilateral blindness due to ocular trauma presenting to the A&E of a teaching hospital eye clinic over a 15-year period | 16 | Male predominance  Most common MOI = chemical assault with car battery acid; bilateral enucleation for ritual purposes and GSW were the next most common | Regulating the accessibility of car battery acid  Community education regarding preventable causes of blindness |
| Gyawali et al., 2017 [(14)](https://www.zotero.org/google-docs/?SclH0h) | Asmara, Eritrea | Low-income | Retrospective case series | Visually impaired pediatric patients (≤ 16 years) admitted presenting to the national referral eye hospital (the only pediatric eye hospital in the country) over a 4-year period | 249 | Trauma was responsible for visual impairment in 34.5% of cases  Blast injury was a notable cause of trauma | Countries impacted by war should continue to work on identifying and eliminating landmines, as they are a preventable cause of blindness |
| Gyawali et al., 2017 [(15)](https://www.zotero.org/google-docs/?btJsRx) | Asmara, Eritrea | Low-income | Cross-sectional study | All children enrolled in the only school for the blind in Eritrea | 92 | Trauma was the second leading cause of blindness in those who had a known cause  Most common cause of ocular trauma = Blast injuries | Wider access to provision of visual correction (i.e. glasses)  Earlier referral to ophthalmologic specialists |
| Hossain et al., 2024 [(76)](https://www.zotero.org/google-docs/?Vvw268) | Sherpur, Bangladesh | Lower middle-income | Prospective observational study | Adult and pediatric patients presenting to a district hospital with ocular trauma over a 6-month period | 100 | Male predominance  11–20-year age group most commonly affected  Most common location of injury = work  Most common MOI = sharp objects  OGI more common than closed globe injuries | None provided |
| Irawati et al., 2024 [(77)](https://www.zotero.org/google-docs/?wBFyae) | IGATES Registry* - data included from 3 LMICs (India, Indonesia, and Nepal) | India = lower middle-income  Indonesia = upper middle-income  Nepal = lower middle-income | Retrospective review of an ocular trauma registry | All patients with sports-related trauma | 322 | Male predominance  Ages 20-39 had the highest incidence of sports-related ocular trauma  Closed globe injuries were more common than OGI  Only 2 patients were wearing eye protection | Better education on the importance of wearing eye protection during sports |
| Islam et al., 2017 [(66)](https://www.zotero.org/google-docs/?dUtebb) | Chittagong, Bangladesh | Lower middle-income | Mixed methods - Retrospective case series and community survey | Retrospective case series: all patients admitted to the hospital with ocular trauma over a 14-month period  Community survey: patients with ocular injuries living in the community, who were invited for a free check-up via mass publicity | 426 (chart review), 126 (community survey) | OGI was the most common cause of admission on Retrospective case series  The majority of community dwellers with ocular injury had CGI  20% of women injured in the community were assaulted by their husbands  Community dwellers reported being prescribed topical steroids by village physicians | Better education for village physicians and the general community about avoidance of topical steroids and undergoing proper evaluation after ocular trauma |
| Jac‑Okereke et al., 2021[(38)](https://www.zotero.org/google-docs/?qoqFK7) | Enugu, Nigeria | Lower middle-income | Prospective cross‑sectional descriptive study | Consecutive patients presenting to eye clinics and emergency units at two hospitals with ocular trauma over a 5-month period | 81 | Male predominance  Ages 10-19 had the highest incidence of ocular trauma  Most common location of injury = home  Most common MOI = blunt implements  Majority of injuries were CGI | None provided |
| Jha et al., 2019 [(39)](https://www.zotero.org/google-docs/?8tkL9l) | Pondicherry, India | Lower middle-income | Prospective case series | All patients presenting to a rural tertiary care center with ocular injury over a 3-year period | 129 | Male predominance  Most common MOI = RTA  Majority of cases were CGI  25.8% of patients (all male) were intoxicated at the time of presentation  Minority of patients were using eye protection at the time of injury | Strengthening legislation and law enforcement surrounding road traffic  Use of helmets with facial protection for individuals riding two-wheeled vehicles |
| Jovanovic et al., 2016 [(78)](https://www.zotero.org/google-docs/?UCakZ1) | Zenica, Bosnia and Herzegovina | Upper middle-income | Retrospective case series | All patients admitted with ocular injury over a 9-year period | 258 | Agricultural workers were 8 times more likely to experience ocular trauma than manual workers  Majority of injuries were non-occupational | Enforcing occupational eye protection and safety training |
| Kaçer and Kaçer, 2022 [(16)](https://www.zotero.org/google-docs/?dNcoxM) | Aksaray, Türkiye | Upper middle-income | Retrospective case series | Pediatric patients (< 18 years) with ocular trauma presenting to the pediatric ED of a tertiary care hospital over a 4-year period | 202 | Male predominance  7–11-year age group was most commonly affected  Injuries were more common in the summer  Injuries typically occurred in situations where parental supervision was lacking (for example: at playgrounds or weddings)  CGI predominated | Parents need to closely supervise their children, particularly in public and busy settings  Protective goggles should be worn during activities that place the eyes at risk of injury  Parents should secure sharp objects and chemicals in locations that children cannot reach |
| Karim-Zade et al., 2016 [(17)](https://www.zotero.org/google-docs/?FO5HxW) | Tajikistan | Lower middle-income | Retrospective case series | Pediatric patients (< 15 years) admitted to three pediatric ophthalmology hospitals in two regions of Tajikistan over a 2-year period | 454 | Male predominance  Most common injury location = outdoors and in rural settings during unsupervised play  Most common MOI = sharp objects  OGI = majority of cases  Majority of cases presented within 24 hours of injury  25% of injuries resulted in blindness in the affected eye | Public health efforts to educate the public about the risks of ocular trauma |
| Khatry et al., 2004 [(55)](https://www.zotero.org/google-docs/?bvb55I) | Sarlahi district, Nepal | Lower middle-income | Prospective case series | All patients with ocular trauma presenting to the only eye care clinic in the district over a 5-  year period | 525 | Male predominance  Mean age 28 years  The mean time between injury and seeking eye care at any location was 7 days; delay was associated with worse VA outcomes | Better education of rural non-ophthalmologists regarding the management of minor ocular injuries  Public education regarding ocular injury prevention |
| Krishnaiah et al., 2006 [(69)](https://www.zotero.org/google-docs/?DtXUwj) | Hyderabad, India | Lower middle-income | Population-based cross-sectional epidemiological study | Individuals of all ages representative of the rural population of southern India | 7771 (824 with ocular trauma) | Odds ratio for ocular trauma was higher for males and laborers  Most injuries were occupational and the majority of those impacted were not wearing eye protection at the time of injury  Most common MOI = plant matter (i.e. thorns, branches, etc.) | None provided |
| Kyei et al., 2023 [(51)](https://www.zotero.org/google-docs/?OLlYUT) | Harare, Zimbabwe | Lower middle-income | Retrospective cross-sectional study | Patients presenting to an outpatient department with ocular trauma over a 4-year periods | 863 | Male predominance  Ages 18-35 years had the highest incidence of ocular trauma  OGI made up the largest proportion of injuries  OGI patients were 10 times more likely to be blinded  Most common MOI = blunt injury | Public education regarding the importance of eye protection and early presentation to the hospital |
| Lama et al., 2022 [(18)](https://www.zotero.org/google-docs/?euwDVk) | Donka, Guinea | Lower middle-income | Prospective longitudinal study | Pediatric patients (≤ 16 years) presenting to a specialized ophthalmology center over a 1-year period | 205 | Male predominance  Ages 5-8 years had the highest incidence of ocular trauma  Most common MOI = Games and sports  Average time from injury to consultation was 3.5 days | Greater parental supervision  Public education on the importance of earlier presentation after ocular trauma |
| Limbu et al., 2018 [(70)](https://www.zotero.org/google-docs/?bpWSXb) | Hetauda, Nepal | Lower middle-income | Community based cross-sectional prospective survey | Workers at 16 different manufacturing sites in the community over a 10-month period | 1236 (473 reported work-related ocular trauma) | The majority of workers never wore protective eyewear at work  Those with prior work-related injuries and who had attended school were more likely to wear protection  Females were less likely to wear eye protection than males | None provided |
| Logday, 2023 [(33)](https://www.zotero.org/google-docs/?LWeeoq) | Cape Town, South Africa | Upper middle-income | Retrospective cohort study | Adult (≥ 18 years) ocular trauma cases presenting to a trauma unit over a 6-month period | 47 | Male predominance  Ages 18-30 years had the highest incidence of ocular trauma  Most common MOI = assault (77%)  OGI = most common injury type  There was a decline in ocular trauma cases during the pandemic | Decreasing the availability of alcohol and illicit substances that make individuals more prone to violence  Restricting access to weapons  Promoting gender equality  Continuing to breakdown societal constructs that promote violence  Encourage people to still seek care for ocular emergencies during future pandemics (though this may be difficult due to lack of transportation during lockdowns) |
| Madan et al., 2020 [(19)](https://www.zotero.org/google-docs/?zvRTTk) | Maharashtra, India | Lower middle-income | Prospective case series | Pediatric patients (≤ 15 years) presenting to an outpatient clinic or ED with ocular trauma | 66 | Male predominance  Ages 6-10 had the highest incidence of ocular trauma  OGI = most common injury type  Most common MOI= sports  18% of children were injured by firecrackers  Nearly half (44%) experienced their ocular trauma while on vacation | Increased parental supervision, particularly during sporting activities  Increased regulations and public education surrounding firecrackers  Implementation of a rapid-action ocular trauma team to address faster management of ocular trauma and educate the public |
| Malik et al., 2011 [(20)](https://www.zotero.org/google-docs/?WPWJm4) | Peshawar, Pakistan | Lower middle-income | Prospective case series | Pediatric patients (1-15 years) with accidental ocular trauma leading to anterior segment injury presenting to the ED or outpatient ophthalmology department over a 16-month period | 200 | Male predominance  Ages 6-10 years had the highest incidence of ocular trauma  Most common MOI = blunt objects  There were significant delays in seeking care | Improving population literacy  Improving population knowledge about ocular trauma prevention  Increased accessibility of ophthalmologic care  More parental supervision |
| Madhusudhan et al., 2014 [(47)](https://www.zotero.org/google-docs/?NrSCuq) | Kota Bharu, Malaysia | Upper middle-income | Retrospective case series | All patients with OGI presenting to the ophthalmology department of a hospital over a 10-year period | 220 | Male predominance  Ages 16-25 had the highest incidence of ocular trauma  Most common injury location= home  Injuries at home were most common among those < 16 years; occupational injuries were the most common among those ≥ 16 years  Most common MOI = sharp objects | Education of the public regarding ocular safety both at work and at home  Development of occupational health and safety guidelines |
| Mansouri et al., 2009 [(79)](https://www.zotero.org/google-docs/?LjmEi5) | Tehran, Iran | Upper middle-income | Retrospective case series | All patients presenting to the ED with OGI over a 5-year period | 2340 | Male predominance  Mean age was 22.4 years, median 19 years  Knife injury was the most common MOI in children, and projectile metallic FB in adults  Severity of ocular injury predicted visual acuity | Family and teacher education regarding the danger of sharp objects  Use of protective eyewear in the workplace and during recreation |
| Maurya et al., 2015 [(21)](https://www.zotero.org/google-docs/?byT5Ur) | Varanasi, India | Lower middle-income | Prospective case series | Pediatric patients (≤ 16 years) presenting to the outpatient ophthalmology department or ED of a tertiary care center over a 3-year period | 82 | Male predominance  Ages 5-10 years had the highest incidence of ocular trauma  Most common site of injury = home  Most common MOI = Projectile objects  A small percentage of patients were injured by fireworks  OGI = most common injury type | Increased parental supervision  Improving population knowledge about ocular trauma prevention, ideally via media  Legislation to ban the use of fireworks and weapons by children |
| Maurya et al., 2019 [(80)](https://www.zotero.org/google-docs/?lk6mmm) | Uttar Pradesh, India | Lower middle-income | Prospective case series | All patients presenting to the outpatient ophthalmology department or ED of a teaching hospital over a 4-year period | 402 | Male predominance  Ages 6-15 years had the highest incidence of ocular trauma  Injuries were more common in the summer season  Most common MOI = non-occupational; sporting accidents followed by RTA  The majority of patients were not wearing eye protection at the time of injury | Public education about risks and prevention of ocular trauma (particularly the use of safety glasses during high-risk activities) |
| Megbelayin et al., 2016 [(40)](https://www.zotero.org/google-docs/?DJrnad) | Calabar, Nigeria | Lower middle-income | Retrospective case series | All patients presenting with ocular trauma to the outpatient eye department of a teaching hospital over a 1-year period | 104 | Male predominance  Ages 21-30 years had the highest incidence of ocular trauma  Most common MOI = assault  The vast majority of cases were CGI caused by blunt trauma  The majority of patients presented 2-7 days after injury | Public education about ocular trauma and prevention strategies (particularly the use of protective eyewear)  Development of a more efficient referral system for ocular injury patients |
| Mokhles and Khalid, 2024 [(81)](https://www.zotero.org/google-docs/?I3TQsR) | Basrah, Iraq | Upper middle-income | Retrospective case series | All patients presenting to the ophthalmologic ED at a teaching hospital over a 1-year period | 250 (70 with ocular trauma) | Male predominance  Traumatic injuries peaked in the summer  Traumatic ocular injury has increased over time (by 12% compared to historical data) | Public education campaigns targeting ocular injury prevention measures, especially the use of eye protection  Violence reduction programs |
| Movahedinejad et al., 2016  [(67)](https://www.zotero.org/google-docs/?sJVXBf) | Kashan, Iran | Upper middle-income | Retrospective cross-sectional study | All patients admitted due to ocular trauma over a 2.5-year period | 200 | Male predominance  Ages 20-39 years had the highest incidence of ocular trauma  Urban residents and those involved in manual/industrial labor were at higher risk for ocular injury  Most injuries in children occurred during play/sporting activities  Most injuries in adults occurred in the workplace | Public education campaigns targeting ocular injury prevention measures, especially the use of eye protection, and particularly in the workplace |
| Mowatt et al., 2012 [(34)](https://www.zotero.org/google-docs/?qoXcUt) | Kingston, Jamaica | Upper middle-income | Retrospective case series | Adult patients (≥ 17 years) admitted with ocular trauma over a 6-year period | 252 | Male predominance  Median age 32 years  Most common site of injury = home  Most common MOI = RTA followed by nail hammering  Females were more commonly injured with chemicals compared to males; 50% of those cases were due to domestic disputes | None provided |
| Murithi et al., 2008 [(22)](https://www.zotero.org/google-docs/?lIKFkj) | Nairobi, Kenya | Lower middle-income | Retrospective case series | Pediatric patients (≤ 15 years) admitted with ocular trauma over a 4-year period | 182 | Male predominance  Bimodal peaks at 4 and 7 years of age  Most common MOI = sticks  OGI = most common injury type  The minority of patients who were referred to the hospital received analgesics, antibiotics, tetanus toxoid, or eye pads | Public injury prevention programs  Improved resource accessibility at smaller referral facilities |
| Ojabo et al., 2011 [(52)](https://www.zotero.org/google-docs/?ZeCKpF) | Benue State, Nigeria | Lower middle-income | Retrospective case series | All patients presenting to an eye unit with ocular injury over a 5-year period | 1560 | Male predominance  Peak incidence was in the 2nd and 3rd decades of life  Largest proportion of the sample population were farmers  75% presented > 24 hours after injury | Healthcare workers at the primary and secondary levels should be educated about which cases of ocular injury need referral to a tertiary care center  Education on the use of helmets with face shields for drivers and protective eyewear for high-risk occupations  Community education regarding the importance of early presentation for eye injury |
| Onakpoya et al., 2010 [(41)](https://www.zotero.org/google-docs/?MdXfdu) | Nigeria | Lower middle-income | Retrospective case series | Geriatric patients (≥ 65 years) seen for ocular trauma at 4 hospitals in southwestern Nigeria over a 6-year period | 78 | Male predominance  Farms, followed by homes, were the most common locations of injury  Most common MOI = Farming accidents followed by assaults  Assault was a more common MOI in females than in males  Most injuries were CGI | Prevention measures in the workplace (though the authors did not provide specific suggestions) |
| Peleja et al., 2022 [(56)](https://www.zotero.org/google-docs/?JheirX) | Brasília, Brazil | Upper middle-income | Retrospective case series | All patients with traumatic OGI who received emergent surgical treatment in a public tertiary hospital over a 4-year period | 359 | Male predominance  Average age of 32.7 years  Average time between injury and hospital admission was 75.7 hours  Delays of > 72 hours between injury and surgery were associated with poor prognosis | Community education about the importance of early presentation for ocular injury  Workplace compliance with federal regulation ensuring that employees use safety equipment |
| Qadir et al., 2019 [(23,43)](https://www.zotero.org/google-docs/?NSwXk9) | Peshawar, Pakistan | Lower middle-income | Retrospective case series | Pediatric patients (≤ 16 years) admitted with ocular trauma over a 3-year period | 658 | Male predominance  Ages 5-10 years had the highest incidence of ocular trauma  Most common injury location = home  OGI = most common injury type | Greater education of parents, especially those with limited care access and low education levels  Greater parental supervision |
| Qayum et al., 2018 [(24)](https://www.zotero.org/google-docs/?N99gw3) | Jammu, India | Lower middle-income | Retrospective case series | Pediatric patients (≤ 16 years) with ocular trauma presenting to the ED or outpatient department of a tertiary hospital over a 1-year period | 357 | Male predominance  Ages 2-6 years had the highest incidence of ocular trauma  Most common injury location = home  Majority of injuries were CGI  Fingernails or fists were the most common MOI among patients with CGI, and pens among patients with OGI | Greater parental supervision  Use of protective eyewear during high-risk activities  Keeping sharp objects and chemicals out of reach of children |
| Qi et al., 2015 [(44)](https://www.zotero.org/google-docs/?zOC6Ey) | Zhengzhou, China | Upper middle-income | Retrospective case series | All patients admitted for ocular trauma over a 6-year period | 5799 | Male predominance  Ages 45-59 years had the highest incidence of ocular trauma  Most common MOI = Firework-related injuries and RTA  Peaks in incidence occurred around the Chinese New Year and in the summer  Majority of patients presented within 24 hours of injury  OGI = most common injury type | Parents should closely supervise children who are lighting or watching fireworks  Children should not be allowed to set off large fireworks  Schools should ensure safe handling of sharp objects  Use of seatbelts and car seats for children |
| Quayum and Akhanda, 2009 [(82)](https://www.zotero.org/google-docs/?4UgenB) | Mymensing, Bangladesh | Lower middle-income | Prospective case series | All patients admitted with ocular trauma over a 2-year period | 97 | Male predominance  Ages 21-40 had the highest incidence of ocular trauma  Majority of injuries were accidental and occupational in nature  Most common MOI = sharp implement | Adoption of protective eyewear in the workplace  Design of more comfortable protective eyewear  Education regarding ocular safety |
| Rafindadi et al., 2013 [(57)](https://www.zotero.org/google-docs/?tUC1Kg) | Zaria, Nigeria | Lower middle-income | Retrospective case series | All patients treated for ocular trauma at the eye clinic of a teaching hospital over a 2-year period | 142 | Male predominance  Ages 16-30 years had the highest incidence of ocular trauma  Most common location of injury = home  Majority of injuries were minor  Students experienced the greatest proportion of injuries (the authors remarked that there was very little industrial work in the area) | Enforcement of RTA safety regulations  Improvement of playground safety  Legislation on the use of protective eyewear in the workplace |
| Saka es et al., 2017 [(48)](https://www.zotero.org/google-docs/?xsCpNY) | Kebbi, Nigeria | Lower middle-income | Retrospective case series | All patients with traumatic corneal lacerations presenting to an eye clinic of a hospital over a 1-year period | 32 | Male predominance  Most common location of injury = home  Most common MOI = sticks, followed by motorcycle accidents  Majority of patients presented in a delayed fashion | Public education on ocular trauma prevention |
| Serrano et al., 2003 [(25)](https://www.zotero.org/google-docs/?WpUq0w) | Santander, Columbia | Upper middle-income | Retrospective case series | Pediatric patients (≤ 15 years) presenting to the ED for ocular trauma over a 5-year period | 393 | Male predominance  Most common location of injury = home, followed by roads  CGI predominated  Majority of patients with CGI had no visual impairment; more than half of patients with OGI experienced severe visual impairment or blindness | Improved adult supervision  Restriction of pediatric access to sharp objects  Public awareness of the importance of presenting early for eye injury evaluation  Education of primary healthcare providers regarding basic ophthalmologic injury management/proper referrals  Elimination of firework sales to the public  Better enforcement of safety seat and seatbelt use |
| Sharifi et al., 2023 [(26)](https://www.zotero.org/google-docs/?za4Qmk) | Kerman, Iran | Upper middle-income | Cross-sectional study | Pediatric patients (< 18) over a 17-month period | 80 | Male predominance  Patients < 6 years followed by ages 6-12 years had the highest incidence of ocular trauma  Most incidents occurred in the spring and indoors  Most common MOI = sharp objects  Most common injury was globe laceration | Improved supervision of children  Parental education on ocular injury prevention |
| Shtewi et al., 1999 [(53)](https://www.zotero.org/google-docs/?LfCAsb) | Tripoli, Libya | Upper middle-income | Prospective case series | Patients admitted with ocular trauma due to RTA over a 2-year period | 248 | Male predominance  Majority of patients were adults  Majority of patients ≤ 33 years  Majority of patients presented within 24 hours  Majority were not wearing seatbelts at the time of injury | Compulsory seat belt use  Laminated glass windscreens in all vehicles  Avoidance of seating children on parents’ laps in the car  Public education about rules of the road  Use of plastic eyeglasses instead of glass  Better road markings and maintenance |
| Singh et al., 2017 [(27)](https://www.zotero.org/google-docs/?ndeweE) | Madhya Pradesh, India | Lower middle-income | Prospective case series | Pediatric patients (≤ 16 years) presenting to the ED or outpatient department with ocular trauma over a 1-year period | 220 | Male predominance  Ages 6-10 had the highest incidence of ocular trauma  Majority of patients were from rural settings and of lower SES  Most common MOI = sharp organic objects  OGI = most common injury type | Educating caretakers, children, and parents about potential causes of ocular trauma, injury prevention, and the importance of early presentation for medical care |
| Soliman and Macky, 2008 [(45)](https://www.zotero.org/google-docs/?Tol4U2) | Cairo, Egypt | Lower middle-income | Prospective cohort study | All patients admitted for ocular trauma over a 6-month period | 147 | Male predominance  Average age was 22 years  Most injuries occurred in the afternoon  Most common MOI = sharp objects  OGI = most common injury type | Education about protective eyewear, especially in the workplace  Restricting access to firearms and other weapons |
| Soni et al., 2015 [(60)](https://www.zotero.org/google-docs/?GyAgUV) | Peshawar, Pakistan | Lower middle-income | Retrospective case series | All patients with ocular trauma presenting to an ocular OPD over a 4-year period | 3218 | Majority of patients were young adult males  Most common injury location = workplace  Most common MOI = corneal foreign body  Injuries in females primarily occurred at home  Late presentations and attempts by patients to remove corneal foreign bodies themselves were noted to lead to corneal infection | Laws mandating the use of protective goggles in work settings  Improved supervision of children |
| Soong et al., 2008 [(62)](https://www.zotero.org/google-docs/?jZa5Uv) | Kuala Lumpur, Malaysia | Upper middle-income | Prospective case series | All patients presenting to the ophthalmology department of a teaching hospital over a 1-year period | 546 | Male predominance  Mean age 31.5 years  Injuries were most commonly work-related, followed by RTA and domestic accidents  Most common MOI=Power tools  Minority of those with work-related injuries were wearing eye protection at the time of the injury | Mandates surrounding the use of protective eyewear in the workplace  Development of an ocular injury database for Malaysia to further identify opportunities for injury prevention  Improved public education regarding ocular safety  Seatbelt and helmet legislation for drivers  Availability of protective eyewear in “do-it-yourself” stores |
| Stuart et al., 2022 [(35)](https://www.zotero.org/google-docs/?EEi3Wx) | Kimberley, South Africa | Upper middle-income | Retrospective case series | Adult patients (≥ 18 years) admitted or presenting to the clinic of a public ophthalmic referral center over a 1 year period | 240 | Majority of patients were young adult males  Most common MOI = assault; occurred in the home during weekend evenings  There was a significant association with alcohol use  Most victims knew their assailants as acquaintances or friends  Most accidental injuries took place on weekdays during working hours  Majority of injuries were CGI  The minority of patients reached the ophthalmic referral center within 24 hours of injury | Public efforts to restrict risky drinking and interpersonal violence  Eye protection during high-risk activities  Healthcare system efforts to improve ophthalmologic care access/transportation to such care |
| Sukati and Hansraj, 2013 [(58)](https://www.zotero.org/google-docs/?8opG2E) | KwaZulu-Natal, South Africa | Upper middle-income | Retrospective cohort study | All patients with ocular trauma presenting to two selected rural provincial hospitals over a 4-year period | 220 | Male predominance  Ages 21-30 had the highest incidence of ocular trauma  Most common MOI = assault via blunt trauma  Most common injury location = home  OGI = most common injury type | Improving population knowledge about ocular trauma prevention, ideally via media |
| Ter Wei et al., 2024 [(54)](https://www.zotero.org/google-docs/?s0T6Js) | Kuala Lumpur, Malaysia | Upper middle-income | Retrospective case series | All patients undergoing emergent eye surgery for ocular trauma over an 8 month period | 214 | Male predominance  Ages 21-40 had the highest incidence of ocular trauma  Most common injury location = workplace  OGI = most common injury type | Implementation of ocular health and safety regulations for the workplace |
| Tian et al., 2023 [(28)](https://www.zotero.org/google-docs/?GQxFnE) | Guangdong Province, China | Upper middle-income | Retrospective case series | Pediatric patients (≤16 years) admitted with severe chemical/thermal ocular injury over a 5-year period | 105 | Male predominance  Children 0-5 had the highest incidence of injuries  Majority of children were from rural areas  Peak in incidence around the Chinese New Year  Low maternal education level and household income were significantly associated with worse VA | Public education regarding the importance of improved pediatric supervision in rural areas (especially children from low-income families)  Public education on ocular safety |
| Ugalahi et al., 2023 [(29)](https://www.zotero.org/google-docs/?CAgDpS) | Ibadan, Nigeria | Lower middle-income | Retrospective case series | Pediatric patients (≤ 16 years) presenting to the ED with ocular injury over a 6-year period | 109 | Male predominance  Ages 6-11 years had the highest incidence of ocular trauma  Most common injury location = home, followed by school  Majority of injuries occurred during play  Majority were CGI  Odds of sharp object MOI and severe visual impairment were higher in children 0-5 years | Educational programs for parents about ocular trauma prevention  Educating children about avoidance of throwing objects (except when playing sports)  Improved access to emergency care for the general population |
| Umeh and Umeh, 1997 [(30)](https://www.zotero.org/google-docs/?Bd4qo7) | Enugu, Nigeria | Lower middle-income | Retrospective case series | Pediatric patients (1-15 years) treated for eye injury at the ophthalmology of a teaching hospital over a 2 year period | 228 | Male predominance  Ages 6-10 years had the highest incidence of ocular trauma  Most common injury location = home  Most common MOI = sticks  Minority sought care within 24 hours  Factors contributing to delay included distance to travel, costs, preference for traditional healing, healthcare workers failing to refer | Increased parental supervision  Community education about ocular injury prevention  Increased healthcare resources for treating ocular trauma in the community |
| Wang et al., 2012 [(36)](https://www.zotero.org/google-docs/?MYZ88b) | Greater Beijing, China | Upper middle-income | Prospective cohort study | Adult patients (≥ 40 years) in the community who underwent a detailed ocular exam; prospective information on ocular trauma was collected at a 5-year repeat visit | 4439 (72 with ocular trauma) | Male gender, rural residence, and alcohol use were associated with ocular trauma  Odds ratio of ocular trauma decreased with increasing age  Prevalence of ocular trauma was 1.6%  5-year incidence of ocular trauma in the population was 2.6% | None provided |
| Wang et al., 2017 [(61)](https://www.zotero.org/google-docs/?zoYlO8) | Hunan, China | Upper middle-income | Retrospective case series | All patients admitted with ocular trauma over a 5-year period | 2009 | Male predominance  Ocular trauma peaked between 41-50 years  Most common location of injury = Workplace in adults, and the home among children  OGI = most common injury type  Firework-associated trauma peaked around the time of the Chinese New Year | Outreach programs in rural areas (particularly through schools) to educate pediatric patients and families on ocular trauma prevention  Mandating the use of protective eyewear in the workplace  Better community education and public legislation to reduce firework injuries |
| Wei et al., 2022 [(46)](https://www.zotero.org/google-docs/?l0ltkd) | Srinagar, India | Lower middle-income | Retrospective case series | Patients admitted to a tertiary hospital with pellet gun–related ocular injuries over a 4-month period** | 777 | Almost all patients were male (97.7%)  Ages 20-29 had the highest incidence of ocular trauma  OGI = most common injury type | Civilian education on the use of protective eyewear in regions where pellet guns are being used heavily by law enforcement officials  Improving access and reducing cost of such eyewear |
| Zhang et al., 2017 [(49)](https://www.zotero.org/google-docs/?7Ua1Hm) | Cangzhou, China | Upper middle-income | Retrospective case series | Patients admitted with ocular trauma over a 4-year period | 478 | Male predominance  Ages 45-59 had the highest incidence of ocular trauma, followed by those 30-44 years  Most common location of injury = workplace, followed by the home  Majority of patients presented the same day as their injury | Public awareness of the importance of seeking early medical care for ocular trauma  Educate the public about using protective eyewear in the workplace and at home during high-risk activities |
| Zhang et al., 2021 [(83)](https://www.zotero.org/google-docs/?BGbxYW) | Tianjin, China | Upper middle-income | Cross-sectional study | Athletes from Tianjin University of Sports, Tianjin Vocational College of Sports, and Tianjin provincial sports teams over a 6-month period | 1413 (151 with ocular trauma) | Male predominance  Incidence of sports-related ocular injury was 10.7%, and 11.9% had impaired vision  Majority of injuries occurred during training  Most common MOI= handball  Adnexal wounds were the most common injuries identified  Younger age, lower SES and more lengthy practice sessions put patients of increased risk of injury  Majority had a delay in seeking care | Use of protective eyewear in contact sports (and financial support for those who cannot afford it)  Improve awareness of ocular trauma risks, especially in low SES athletes  Ensuring athletes are not overworked during practices |
| Zhi Hong et al., 2020 [(50)](https://www.zotero.org/google-docs/?7VSRBB) | Maharashtra, India | Lower middle-income | Prospective cross-sectional study | Patients from the IGATES* registry presenting to the ED or outpatient department of a tertiary care center over a 2-year period | 208 | Male predominance  Majority of patients were ≤ 40 years  Industrial accidents were the most common activity at the time of injury  Most common MOI= penetrating injury  Wood was the most common implement of injury | Use of protective eyewear in the workplace and during sports |
| Zhou et al., 2021 [(3)](https://www.zotero.org/google-docs/?53idqC) | Handan, China | Upper middle-income | Cross-sectional population-based study | Census of adults (≥ 30 years) in 13 rural villages; participants were invited to attend a county hospital for a detailed ocular examination and interview | 6830 (124 with ocular trauma) | Men at higher risk of ocular trauma (OR 3.3)  Those with a history of falls were at higher risk of ocular trauma (OR 2.4)  10.5% with unilateral visual impairment due to trauma  21% with unilateral blindness due to trauma | Education for rural communities regarding eye health/injury prevention  Appropriate first aid for ocular trauma in rural settings |

A&E = accident and emergency, CGI = closed-globe injury, ED = emergency department, FB = foreign body, GSW = gunshot wound, IOFB = intraocular foreign body, LMIC = low- and middle-income countries, MOI = mechanism of injury, NOI = neuro-ophthalmic injury, OGI = open globe injury, OPD = outpatient department, OR = odds ratio, RTA = road traffic accident, SES = socioeconomic status, VA = visual acuity. *IGATES Registry - International Globe and Adnexal Trauma Epidemiology Study - includes data on ocular trauma from 8 countries (Guatemala, India, Indonesia, Iran, Mexico, Nepal, Pakistan, USA) [(84)](https://www.zotero.org/google-docs/?WMe3Fh). **Pellet guns have been used to deal with public unrest in Kashmir, India; the data for this study was collected during a period of massive public unrest.
