# Supplementary material for: Epidemiology of ocular trauma in limited-resource settings: a narrative review
Source: Front Med (Lausanne). 2025 Aug 25;12:1585527. doi: 10.3389/fmed.2025.1585527 (PMC12415046; doi:10.3389/fmed.2025.1585527)
Supplement: Supplementary file 2 [file Table_2.docx]

**INPUT PROMPT TO MICROSOFT COPILOT 365**

Using supplementary Table 1 (attached), note any major differences across the "key findings" column between the adult and pediatric populations. Please cite references. Supplementary Table 1.docx

**OUTPUT FROM MICROSOFT COPILOT 365**

Based on the content of **Supplementary Table 1**, here are the **major differences in key findings** between **adult** and **pediatric** populations regarding ocular trauma:

**🔹 1. Location and Context of Injury**

- **Pediatric Population**:
  - Injuries most commonly occurred **at home** or during **unsupervised play** (Ahmadabadi et al., 2010 [10]; Al-Bdour and Azab, 1998 [11]; Dulal et al., 2012 [12]; Maurya et al., 2015 [21]).
  - Common mechanisms included **sharp objects**, **sticks**, **sports**, and **fireworks**.
  - Injuries often occurred during **vacation periods** or **festive seasons** (e.g., Chinese New Year) (Tian et al., 2024 [28]; Madan et al., 2020 [19]).
- **Adult Population**:
  - Injuries were more frequently **workplace-related**, especially in **agriculture**, **construction**, and **manufacturing** (Cai and Zhang, 2015 [74]; Chaikitmongkol et al., 2015 [31]).
  - **Assault** and **road traffic accidents (RTAs)** were also common causes (du Toit et al., 2013 [32]; Jha et al., 2019 [39]).

**🔹 2. Mechanism of Injury (MOI)**

- **Pediatric**:
  - **Sharp objects**, **sticks**, **sports equipment**, and **fireworks** were leading causes (Ahmadabadi et al., 2010 [10]; Singh et al., 2017 [27]; Malik et al., 2011 [20]).
  - **Blast injuries** were notable in conflict-affected regions (Gyawali et al., 2017 [14], [15]).
- **Adult**:
  - **Occupational injuries** (e.g., from metal, tools, or chemicals) were predominant (Cai and Zhang, 2015 [74]; Krishnaiah et al., 2006 [69]).
  - **Assault-related trauma** was more common in adults, often associated with **alcohol use** (du Toit et al., 2013 [32]; Stuart et al., 2022 [35]).

**🔹 3. Delay in Presentation**

- **Pediatric**:
  - Delays were often due to **lack of parental awareness**, **distance to care**, or **use of traditional remedies** (Umeh and Umeh, 1997 [30]; Malik et al., 2011 [20]).
- **Adult**:
  - Delays were linked to **weekend injuries**, **visiting non-specialist facilities first**, or **self-treatment** (Al-Attas et al., 2010 [73]; Ajayi et al., 2014 [68]).

**🔹 4. Visual Outcomes**

- **Pediatric**:
  - Higher risk of **severe visual impairment or blindness** due to delayed care and more severe injuries like **OGI** (Serrano et al., 2003 [25]; Ahmadabadi et al., 2010 [10]).
- **Adult**:
  - Visual outcomes were often tied to **severity of injury** and **timing of surgical intervention** (Peleja et al., 2022 [56]; Mansouri et al., 2009 [79]).

**🔹 5. Prevention Recommendations**

- **Pediatric**:
  - Emphasis on **parental supervision**, **safe storage of hazardous items**, **firework regulation**, and **school-based education** (Ahmadabadi et al., 2010 [10]; Maurya et al., 2015 [21]; Kaçer and Kaçer, 2022 [16]).
- **Adult**:
  - Focus on **workplace safety**, **protective eyewear**, **training for temporary workers**, and **legislation enforcement** (Cai and Zhang, 2015 [74]; Chaikitmongkol et al., 2015 [31]; Soong et al., 2008 [62]).
